# Hepatocellular Carcinoma in Mice Affects Neuronal Activity and Glia Cells in the Suprachiasmatic Nucleus

**DOI:** 10.3390/biomedicines12102202

**Published:** 2024-09-27

**Authors:** Mona Yassine, Soha A. Hassan, Lea Aylin Yücel, Fathima Faiba A. Purath, Horst-Werner Korf, Charlotte von Gall, Amira A. H. Ali

**Affiliations:** 1 Institute of Anatomy II, Medical Faculty, Heinrich Heine University, Moorenstraße 5, 40225 Düsseldorf, Germany; mona.yassine@hhu.de (M.Y.); soha.hassan@suezuniv.edu.eg (S.A.H.); leaaylin.yuecel@med.uni-duesseldorf.de (L.A.Y.); fathimafaiba.ambalapurath@med.uni-duesseldorf.de (F.F.A.P.); amira.ali@med.uni-duesseldorf.de (A.A.H.A.); 2 Department of Zoology, Faculty of Science, Suez University, P.O. Box 43221, Suez 43533, Egypt; 3 Department of Biology, University of Fribourg, 1700 Fribourg, Switzerland; 4 Institute of Anatomy I, Medical Faculty, Heinrich Heine University, Moorenstraße 5, 40225 Düsseldorf, Germany; korf@uni-duesseldorf.de; 5 Department of Human Anatomy and Embryology, Faculty of Medicine, Mansoura University, Mansoura 35516, Egypt

**Keywords:** HCC, SCN, circadian system, VIP, AVP, glia, oxidative stress

## Abstract

**Background**: Chronic liver diseases such as hepatic tumors can affect the brain through the liver–brain axis, leading to neurotransmitter dysregulation and behavioral changes. Cancer patients suffer from fatigue, which can be associated with sleep disturbances. Sleep is regulated via two interlocked mechanisms: homeostatic regulation and the circadian system. In mammals, the hypothalamic suprachiasmatic nucleus (SCN) is the key component of the circadian system. It generates circadian rhythms in physiology and behavior and controls their entrainment to the surrounding light/dark cycle. Neuron–glia interactions are crucial for the functional integrity of the SCN. Under pathological conditions, oxidative stress can compromise these interactions and thus circadian timekeeping and entrainment. To date, little is known about the impact of peripheral pathologies such as hepatocellular carcinoma (HCC) on SCN. **Materials and Methods**: In this study, HCC was induced in adult male mice. The key neuropeptides (vasoactive intestinal peptide: VIP, arginine vasopressin: AVP), an essential component of the molecular clockwork (Bmal1), markers for activity of neurons (c-Fos), astrocytes (GFAP), microglia (IBA1), as well as oxidative stress (8-OHdG) in the SCN were analyzed by immunohistochemistry at four different time points in HCC-bearing compared to control mice. **Results**: The immunoreactions for VIP, Bmal1, GFAP, IBA1, and 8-OHdG were increased in HCC mice compared to control mice, especially during the activity phase. In contrast, c-Fos was decreased in HCC mice, especially during the late inactive phase. **Conclusions**: Our data suggest that HCC affects the circadian system at the level of SCN. This involves an alteration of neuropeptides, neuronal activity, Bmal1, activation of glia cells, and oxidative stress in the SCN.

## 1. Introduction

Hepatocellular carcinoma (HCC) is the most common liver cancer worldwide and causes approximately one million deaths annually [1]. Recently, there has been an increasing number of HCC patients globally [2], often diagnosed at advanced stages after serious consequences have occurred due to the absence of specific symptoms and lack of diagnostic markers [3]. Non-specific symptoms of HCC include fatigue and sleep disturbance [4,5]. Generally, cancer-related fatigue (CRF) is caused by multiple central and peripheral factors, including neurotransmitter imbalance, systemic inflammation, and circadian disruption [6] that negatively impacts the patient’s quality of life [5].

The hierarchically organized mammalian circadian system controls rhythms in behavior and physiology, such as activity, sleep, body temperature, hormone release, and food intake [7,8]. It contains a master circadian oscillator that generates an endogenous rhythm of approximately 24 h (circadian), input paths for the entrainment of the circadian rhythms with rhythmic external environmental cues, and output paths for the entrainment of subordinate oscillators. The master circadian oscillator resides in the hypothalamic suprachiasmatic nucleus (SCN) [9]. Rhythmic SCN activity is entrained to the light/dark cycle through the retino-hypothalamic tract (RHT). The integrated time information is transmitted via neuronal and endocrine connections to subordinate oscillators in the brain and the periphery. The SCN can be divided into two functionally different subregions: the ventrolateral core and the dorsomedial shell. Both regions use GABA as a transmitter but in combination with different neuropeptides, the vasoactive intestinal peptide (VIP) in the core region and arginine vasopressin (AVP) in the shell region [10]. The core is innervated by the RHT and innervates the shell, which in turn innervates other key regions of the hypothalamus [11]. At the cellular level, a molecular clockwork composed of transcriptional/translational feedback loops of clock genes controls rhythmic cell function. The transcription factor Bmal1 is an essential component of molecular clockwork and is involved in the generation and entrainment of circadian rhythms in SCN [12,13]. At the SCN cellular network level, the coupling of both neurons and astrocytes [14,15], as well as VIP and AVP [10,16,17,18,19], contribute to both the stability and the entrainment of circadian rhythms. Light information is encoded by the release of glutamate and neuropeptides from RHT connecting to VIP neurons in the core. The stimulation of the respective receptors activates the MAPK/CRE-binding pathways and, subsequently, the neuronal activity marker c-Fos and clock gene Per1 to reset the molecular clockwork [20,21,22].

So far, little is known about the pathomechanisms of structural and functional changes in the brain caused by chronic liver diseases. The liver–brain axis might involve visceral afferent connections, including sympathetic and parasympathetic nervous systems [23] and blood-borne signals such as immune cells, cytokines, and metabolites that reach the brain, particularly through a disease-related deficient blood–brain barrier [1,23,24]. In hepatic encephalopathy, ammonia and inflammation are the major triggers for oxidative stress, astrocytic/neuronal dysfunction, and subsequent disturbances of brain oscillatory networks [25,26,27]. HCC involves pathologies in liver structure and function associated with chronic inflammation, resulting in hepatocellular DNA damage, and can be recapitulated by the administration of genotoxic agents in animals [28]. HCC is associated with sleep–wake disturbance and poor sleep quality in patients [29]. Higher diurnal cortisol level suggests a disturbed circadian rhythm in HCC patients, which may underlie their poor sleep quality [30].

Clock genes are pivotal players that regulate cell proliferation and apoptosis in HCC [31]. Furthermore, they are considered potential diagnostic markers and therapeutic targets in HCC [32]. HCC affects transcription and posttranscriptional modification of clock genes [32] and, consequently, rhythmic functions also in other tissues [33,34,35], including the hippocampus [36].

In the hippocampus, HCC-dependent changes in clock genes are associated with upregulation of genes encoding for proinflammatory cytokines and reduced proliferation of neuronal progenitors [36], suggesting that liver cancer affects the molecular clockwork, the innate immune system, and adult neurogenesis in the brain. Moreover, we showed earlier that HCC is associated with changes in corticosterone rhythms, locomotor activity (reminiscent of fatigue), and pERK immunoreaction in SCN [37], suggesting that the circadian system is compromised by liver cancer. The aim of this study was to better understand the effect of HCC on the circadian system. We investigated the neuropeptides, SCN neuronal and glial activity, molecular clockwork, and oxidative stress in the context of HCC.

## 2. Materials and Methods

### 2.1. Animal Experiments and Tissue Preparation

The animal experiments were approved by the local authority, the Landesamt für Natur, Umwelt und Verbraucherschutz Nordrhein-Westfalen (Reference number: AZ 81-02.04.2018-A146) and were conducted according to Directive 2010/63/EU of European Parliament and the Council on the Protection of Animals Used for Scientific Purposes. The experimental design is described in detail in [35,36,37]. Briefly, 14-day-old male Per2::Luc mice with a C57BL6/J background were randomly divided into an experimental group and a control group. In the experimental group, HCC was chemically induced by a single intraperitoneal injection of the genotoxic agent diethylnitrosamine (10 mg/kg, Cat # N0756-10ML, Sigma Aldrich, Burlington, MA, USA). Phenobarbital (PB) was chronically added to the drinking water (0.05%, Luminal, Desitin, Hamburg, Germany) to enhance HCC development. The control group was only given PB in the drinking water (PB control). At the age of one month, mice were separated from their mothers, and litters were kept in groups of 3–4 mice per cage under 12 h light/12 h dark. During the light phase, the light intensity was 300 lx. Zeitgeber time (ZT) 00 is defined as the time when light was turned on (06:00 am), while ZT12 is defined as the time when light was turned off (06:00 pm). At the age of 7–8 months, mice were sacrificed at four different time points at six-hour intervals, starting at ZT02, thus two hours after lights were on (08:00 am), ZT08: eight hours after lights were on, ZT14: two hours after lights were off and ZT20: eight hours after the lights were off. This provides us with tissue collected at 2 different time points during the early and late light phase (ZT02, ZT08, respectively), and 2 different time points during the early and late dark phase (ZT14, ZT20) to allow the comparison between these time points and phases.

Mice were injected intraperitoneally with a mixture of ketamine (100 mg/kg, Actavis, Ireland) and xylazine (10 mg/kg, Bayer, Leverkusen, Germany), then were intracardially perfused with 0.9% NaCl, followed by 4% formalin, using a peristaltic pump (World Precision Instruments, Sarasota, FL, USA). For mice sacrificed during the dark phase, perfusion and tissue collection were performed exclusively under dim red light.

The livers were inspected for tumors (Figure S1). Of the experimental group, only the mice of comparable tumor size were included (n = 12), while animals with no visible or too small lesions were excluded (n = 4). Of the PB control group, all mice could be included because none had a tumor (n = 12). The brains were extracted, post-fixed in 4% formalin for additional 24 h, cryoprotected in 30% sucrose, and snap frozen. The brains were cryo-sectioned into series of free-floating 30 μm thick coronal sections. Series of sections, including the SCN from these animals, were used for immunohistochemical staining in our previous work [37], while the other series were used here to obtain our novel data published here.

### 2.2. Immunohistochemistry

All sections used for each marker were processed together at one staining set to avoid the variability in staining conditions.

#### 2.2.1. Chromogenic Reaction

Step 1: Serial brain sections were washed with phosphate-buffered saline (PBS). Step 2: Sections were incubated with 0.6% H_2_O_2_ for 30 min, followed by washing with PBS containing 0.2% Triton (PBST). Step 3: Sections were incubated in PBST containing 5% normal goat serum (PBSTN) for one hour, followed by incubation with primary antibodies (Table 1) at 4 °C overnight. Step 4: The sections were incubated with the corresponding IgG-biotin-conjugated secondary antibody (Table 2) in PBSTN for one hour at room temperature. This was followed by washing with PBST and incubation with avidin/biotin-based peroxidase system (ABC kit, Vector Laboratories, Newark, CA, USA) for one hour. The sections were rinsed and, finally, incubated with 0.05% 3,3′-diaminobenzidine (DAB) for 5 min. Step 6: Sections were rinsed with PBS, mounted on slides, and cover slipped using DePeX (Serva Electrophoresis, Heidelberg, Germany).

#### 2.2.2. Immunofluorescence

The steps 1, 3, and 6 were performed as described in Section 2.2.1. Step 2 was omitted. Step 4 was performed as follows: sections were incubated with the corresponding IgG-AlexaFluor-conjugated secondary antibody (Table 2) diluted in PBST containing 5% normal goat serum for one hour at room temperature.

#### 2.2.3. Antibody Specificity

The specificity of primary antibodies has been confirmed previously [38,39,40,41,42,43,44] and was also evident by cell morphology, subcellular localization, and region-specific distribution. The specificity of secondary antibody was confirmed by treating the sections as in Section 2.2.1 and Section 2.2.2 described, but without adding primary antibody. To test the specificity of IgG-biotin-conjugated secondary antibody, sections were incubated with 0.6% H_2_O_2_ for 30 min and then in PBST containing 5% PBSTN at 4 °C overnight. The sections were incubated with IgG-biotin-conjugated secondary antibody in PBSTN for one hour at room temperature, followed by washing and incubation with avidin/biotin-based peroxidase system for one hour. The sections were rinsed and, finally, incubated with DAB for 5 min, then mounted on slides and cover slipped. To test the AlexaFluor-conjugated secondary antibody, the previous steps were repeated without incubation in 0.6% H_2_O_2_. The specificity was confirmed as a positive staining signal was not detected.

### 2.3. Image Acquisition and Analysis

Coronal sections containing the hypothalamus were imaged using ×20 objective of a Keyence BZ-X800 microscope (Keyence, Osaka, Japan). During image acquisition and processing, all settings, including illumination intensity and step size, were kept constant for each staining set. The analysis was performed by an investigator blind to the experimental conditions. Image analysis was performed using ImageJ (Version 1.53a) software [45]. This automated analysis helps obtain standardized and accurate quantification of staining. The SCN was demarcated in the coronal brain sections due to the presence of densely packed nuclei dorsal to the optic chiasm on both sides of the lateral ventricle in the hypothalamic area from Bregma −0.34 to −0.7 [46]. Boundaries were traced using ImageJ’s freehand tool (Figure S2A). The immunoreactions were analyzed in the middle part of the rostrocaudal extent in the right and left SCN from 1 to 2 sections per mouse and then averaged to obtain one value per animal. The intensity of background signal in the adjacent neuropil was subtracted from the respective intensity of immunostaining for VIP, AVP, Bmal1, glial fibrillary acidic protein (GFAP), IBA1, and 8-hydroxydeoxyguanosine (8-OHdG) in the SCN, and the resulting intensity was given in arbitrary units (A.U.). The number of c-Fos-immunoreactive cells in the delineated SCN area was counted and given in cells/mm^2^. The lateral hypothalamus was demarcated in the coronal brain sections due to the presence of orexin-immunoreactive neurons in the area from Bregma −1.22 to −2.18 [46]. Boundaries were traced using ImageJ’s freehand tool (Figure S2B). The number of orexin-immunoreactive cells in the delineated area of the lateral hypothalamus was counted and given in cells/mm^2^.

### 2.4. Statistical Analysis

GraphPad Prism 8 software was used for statistical analysis. Data are expressed as mean ± standard error of the mean (SEM). Two-way analysis of variance (ANOVA) was used to examine the effect of time and HCC and their interaction, followed by Sidak’s multiple comparisons test. The results were regarded as significant when *p* value was <0.05.

## 3. Results

### 3.1. Effect of HCC on Neuropeptides in SCN and Lateral Hypothalamus

**Figure 1:**
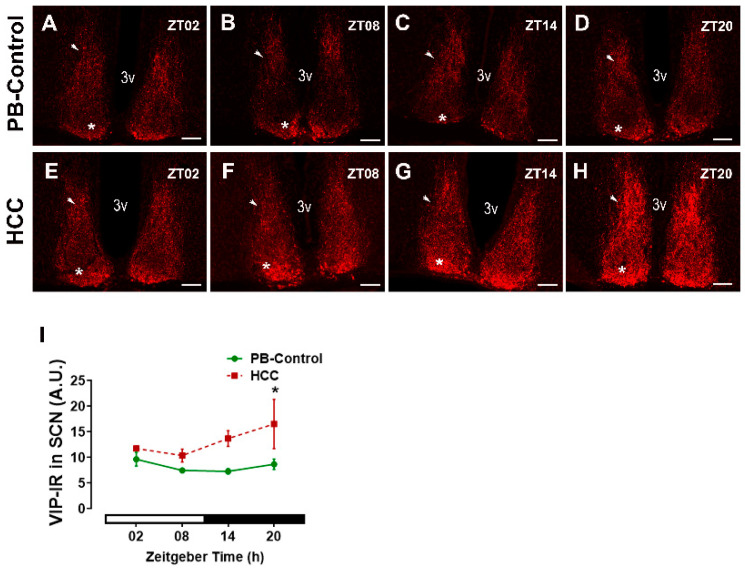
Vasoactive intestinal peptide (VIP) immunoreaction (IR) in the SCN. Representative fluorescent microphotographs showing the immunoreaction (IR) of VIP (red) in the suprachiasmatic nucleus (SCN) of (**A**–**D**) PB control mice and (**E**–**H**) HCC mice. The white asterisk indicates the ventral core region, while white arrowhead indicates the dorsal shell region of SCN. 3v: third ventricle. Scale bar = 100 μm. Mice were sacrificed at different time points at 6 h intervals starting at Zeitgeber time (ZT) 02 = 2 h after the light on. (**I**) Quantification of VIP-immunoreaction (IR) in arbitrary unit (A.U.) in the SCN. White and black bar indicates light/dark phase, respectively. Two-way ANOVA followed by Sidak’s multiple comparisons test. Total of 12 mice per group, n = 3 mice at each time point. *: *p* < 0.05 between PB control and HCC.

VIP is expressed by neurons in the SCN core region and is crucial for synchronization of the neuronal network within the SCN [10]. VIP-immunoreactive neurons were found in the SCN of both groups (Figure 1A–H). Their cell bodies were found primarily in the core region, while their axons extended into the shell region (Figure S3A–C). Two-way ANOVA showed an effect of HCC (F(3,16) = 12.3, *p* = 0.003). Post hoc multiple comparisons showed a significantly higher intensity of VIP-immunoreaction (IR) in HCC mice than in PB controls in the late dark/activity phase (ZT20, *p* = 0.04) (Figure 1D,H,I).

**Figure 2:**
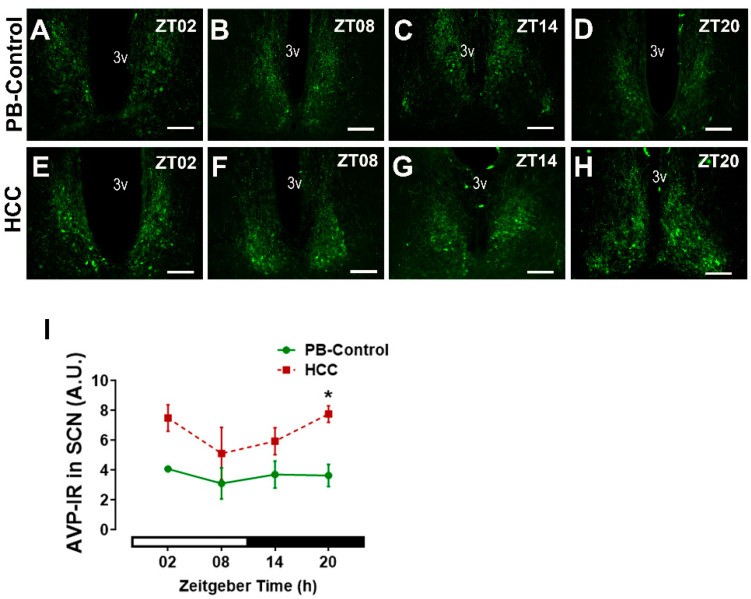
Arginine vasopressin (AVP) immunoreaction (IR) in the SCN. Representative fluorescent microphotographs showing the immunoreaction (IR) of AVP (green) in the SCN of (**A**–**D**) PB control mice and (**E**–**H**) HCC mice. 3v: third ventricle. Scale bar = 100 μm. Mice were sacrificed at different time points at 6 h intervals starting at ZT02 = 2 h after the light was on. (**I**) Quantification of AVP-immunoreaction (IR) in arbitrary unit (A.U.) in the SCN. White and black bar indicates light/dark phase, respectively. Two-way ANOVA followed by Sidak’s multiple comparisons test. Total of 12 mice per group, n = 3 mice at each time point. *: *p* < 0.05 between PB control and HCC.

AVP is also a synchronizer for SCN neurons and plays an important role in determining the period length and the anticipatory behavior [10]. AVP-immunoreactive neurons and axons were found in SCN of both groups, mainly in the shell regions (Figure 2A–H and Figure S3D,E). Two-way ANOVA showed an effect of HCC (F(3,16) = 18.7, *p* = 0.0005) (F(3,16) = 0.5, *p* = 0.66). Post hoc multiple comparisons showed a significantly higher intensity of AVP-IR in HCC mice than in PB controls in the late dark/activity phase (ZT20, *p* = 0.03) (Figure 2D,H,I).

**Figure 3:**
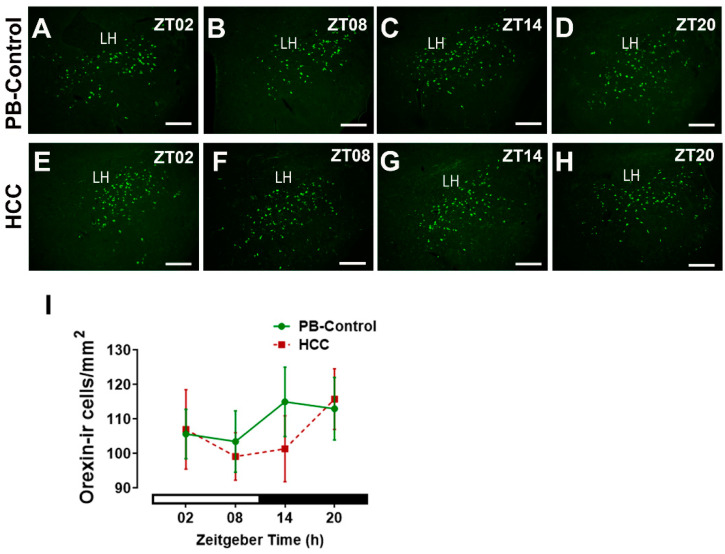
Orexin-immunoreactive (ir) cells in the lateral hypothalamus. Representative fluorescent microphotographs showing orexin-ir cells (green) in the lateral hypothalamus (LH) of (**A**–**D**) PB control mice and (**E**–**H**) HCC-bearing mice. Scale bar = 200 μm. Mice were sacrificed at different time points at 6 h intervals starting at ZT02 = 2 h after the light was on. (**I**) Quantification of number of orexin-immunoreactive cells per mm^2^ in the lateral hypothalamus. White and black bar indicates light/dark phase, respectively. Two-way ANOVA followed by Sidak’s multiple comparisons test. Total of 12 mice per group, n = 3 mice at each time point.

Orexin-immunoreactive neurons in the lateral hypothalamus play an essential role in the control of the sleep–wake cycle and the locomotor activity downstream of SCN. Two-way ANOVA showed no effect of HCC (F(3,16) = 0.7, *p* = 0.59) on the number of orexin-immunoreactive neurons in the lateral hypothalamus (Figure 3A–I).

### 3.2. Effect of HCC on Bmal1-Immunoreactive Cells in SCN

**Figure 4:**
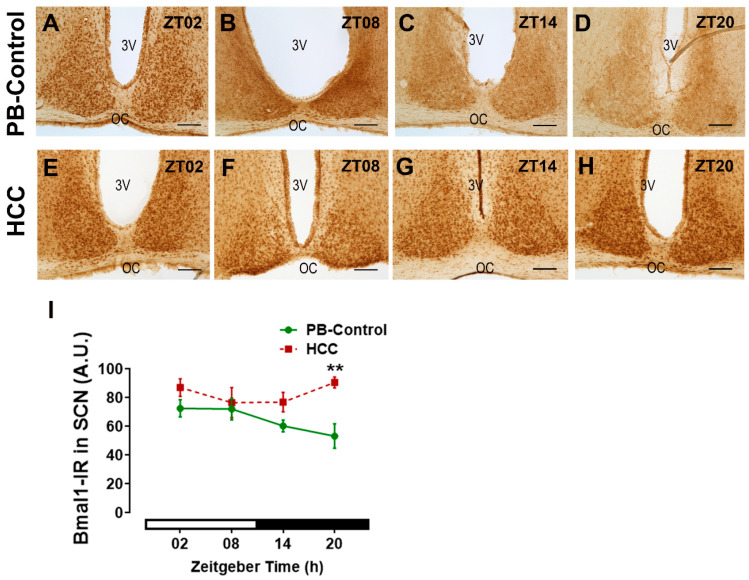
Bmal1-immunoreaction (IR) in the SCN. Representative bright-field photomicrographs showing the Bmal1-immunoreactive cells (brown staining) in the SCN of (**A**–**D**) PB control mice and (**E**–**H**) in HCC-bearing mice. 3v: third ventricle. OC: optic chiasma. Scale bar = 100 μm. Mice were sacrificed at different time points at 6 h intervals starting at ZT02 = 2 h after the light on. (**I**) Quantification of Bmal1-IR in arbitrary units (A.U.) in the SCN at individual time points at 6 h intervals. White and black bar indicates light/dark phase, respectively. Two-way ANOVA followed by Sidak’s multiple comparisons test. Total of 12 mice per group, n = 3 mice at each time point. **: *p* < 0.01 between PB control and HCC.

Immunoreactive cells for the essential circadian clock protein Bmal1 (Figure 4A–H) showed no differences in the distribution between the two SCN subregions. Two-way ANOVA showed an effect of HCC (F(3,16) = 13.7, *p* = 0.002) (F(3,16) = 2.0, *p* = 0.16). Post hoc multiple comparisons showed a significantly higher intensity of Bmal1-IR in HCC mice than in PB controls in the late dark/activity phase (ZT20, *p* = 0.006) (Figure 4D,H,I).

### 3.3. Effect of HCC on the Neuronal Activity Marker c-Fos in SCN

**Figure 5:**
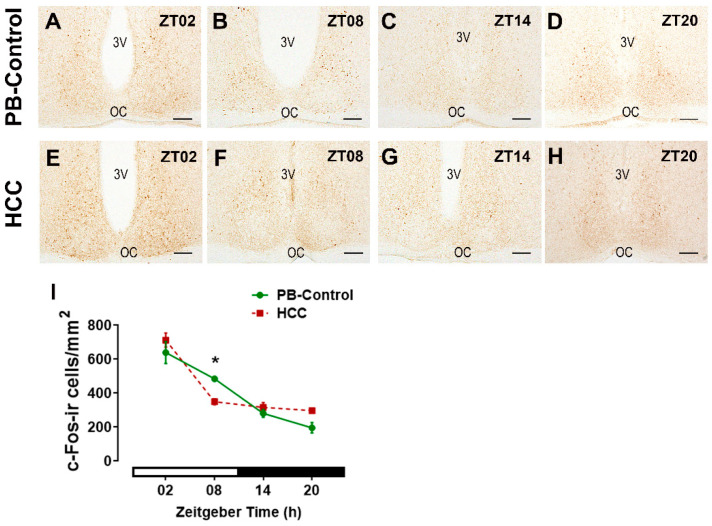
c-Fos-immunoreactive (ir) cells in the SCN. Representative bright-field photomicrograph showing the positively stained c-Fos cells (brown staining) in the SCN of (**A**–**D**) PB control mice and (**E**–**H**) in HCC-bearing mice. 3v: third ventricle. OC: optic chiasma. Scale bar = 100 μm. Mice were sacrificed at different time points at 6 h intervals starting at ZT02 = 2 h after the light was on. (**I**) Quantification of c-Fos-ir cells/mm^2^ in the SCN. White and black bar indicates for light/dark phase, respectively. Two-way ANOVA followed by Sidak’s multiple comparisons test. Total of 12 mice per group, n = 3 mice at each time point. *: *p* < 0.05 between PB control and HCC.

The immunoreaction of c-Fos, the neuronal activity marker, was found in SCN cells of both groups (Figure 5A–H). Two-way ANOVA showed an interaction between time of day and HCC (F(3,16) = 5.2, *p* = 0.02). Post hoc multiple comparisons showed a significantly lower number of cFos-immunoreactive cells in HCC mice than in PB controls in the late light/inactive phase (ZT08, *p* = 0.04) (Figure 5B,F,I).

### 3.4. Effect of HCC on Astrocytic Marker GFAP in SCN

**Figure 6:**
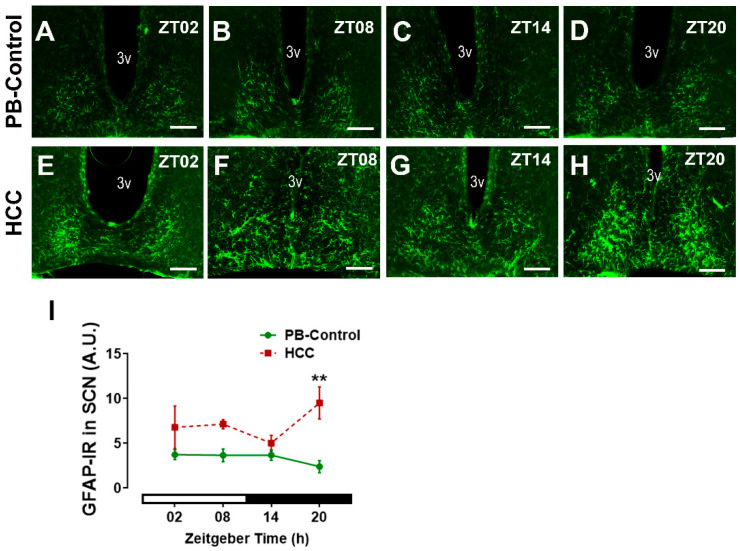
Astrocytic marker GFAP-immunoreaction (IR) in SCN. Representative fluorescent microphotographs showing GFAP immunoreaction (IR) (green) in the SCN of (**A**–**D**) PB control mice and (**E**–**H**) HCC mice. 3v: third ventricle. Scale bar = 150 μm. Mice were sacrificed at different time points at 6 h intervals starting at ZT02 = 2 h after the light was on. (**I**) Quantification of GFAP-immunoreaction (IR) in arbitrary unit (A.U.) in the SCN. White and black bar indicates light/dark phase, respectively. Two-way ANOVA followed by Sidak’s multiple comparisons test. Total of 12 mice per group n = 3 mice at each time point. **: *p* < 0.01 between PB control and HCC.

To investigate if HCC affects astrocytes in the SCN, we used the marker of astrocytic processes GFAP, whose increase within brain tissue indicates reactive astrocytes [47] (Figure 6A–H). Two-way ANOVA showed an effect of HCC (F(3,16) = 19.6, *p* = 0.0004). Post hoc multiple comparisons showed a significantly higher intensity of GFAP-IR in HCC mice than in PB controls in the late dark/active phase (ZT20, *p* = 0.003) (Figure 6D,H,I).

### 3.5. Effect of HCC on Microglial Marker IBA-1 in SCN

**Figure 7:**
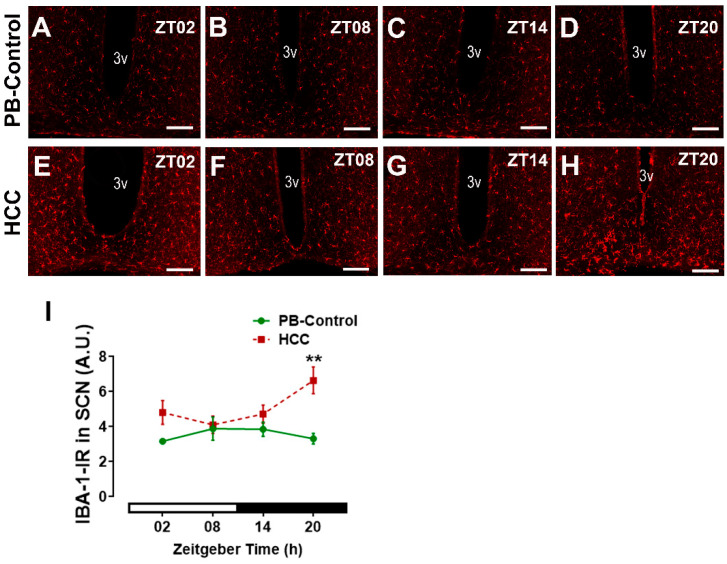
Microglial marker IBA-1-immunoreaction (IR) in SCN. Representative fluorescent photomicrographs showing IBA-1-immunoreaction (IR) (red) in the SCN of (**A**–**D**) PB control mice and of (**E**–**H**) HCC-bearing mice. 3v: third ventricle. Scale bar = 150 μm. Mice were sacrificed at different time points at 6 h intervals starting at ZT02 = 2 h after the light was on. (**I**) Quantification of IBA-1-immunoreaction (IR) in arbitrary unit (A.U.) in the SCN. White and black bar indicates for light/dark phase, respectively. Two-way ANOVA followed by Sidak’s multiple comparisons test. Total of 12 mice per group, n = 3 mice at each time point. **: *p* < 0.01 between control and HCC.

We analyzed whether HCC induced microglial activation in SCN as an inflammatory response by use of IBA-1, the most commonly used marker for microglia [48]. IBA-1-immunoreactive microglia were detected in the SCN of PB control mice (Figure 7A–D) and the HCC-bearing mice (Figure 7E–H). Two-way ANOVA showed an effect of HCC (F(3,16) = 16.2, *p* = 0.001). Post hoc multiple comparisons showed a significantly higher intensity of IBA1-IR in HCC mice than in PB controls in the late dark/active phase (ZT20, *p* = 0.002) (Figure 7D,H,I).

### 3.6. Effect of HCC on Oxidative Stress Marker 8-OHdG in SCN

**Figure 8:**
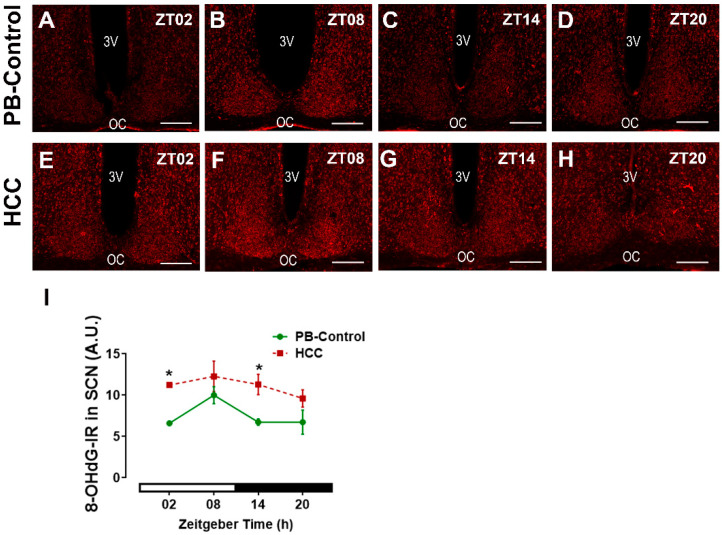
Oxidative stress marker 8-OHdG-immunoreaction in (IR) SCN. Representative fluorescent photomicrographs showing the immunoreaction (IR) of 8- hydroxydeoxyguanosine (8-OHdG) (red) in the SCN of (**A**–**D**) PB control mice and of (**E**–**H**) HCC-bearing mice. 3v: third ventricle. OC: optic chiasma. Scale bar = 100 μm. Mice were sacrificed at different time points at 6 h intervals starting at ZT02 = 2 h after the light was on. (**I**) Quantification of 8-OHdG-immunoreaction (IR) in arbitrary unit (A.U.) in the SCN. White and black bar indicates light/dark phase, respectively. Two-way ANOVA followed by Sidak’s multiple comparisons test. Total of 12 mice per group, n = 3 mice at each time point. *: *p* < 0.05 between PB control and HCC.

To analyze whether HCC induced oxidative stress in SCN, we investigated the DNA oxidative stress marker 8-hydroxydeoxyguanosine (8-OHdG) (Figure 8A–H). Two-way ANOVA showed an effect of HCC (F(3,16) = 21.7, *p* = 0.0003). Post hoc multiple comparisons showed a significantly higher intensity of 8-OHdG-IR in HCC mice than in PB controls in the early light/inactive phase (ZT02, *p* = 0.03) (Figure 8A,E,I) and early dark/active phase (ZT14, *p* = 0.04) (Figure 8C,G,I).

## 4. Discussion

There is increasing evidence of a bidirectional relationship between cancer and the circadian system. On the one hand, cancer patients suffer from sleep–wake disturbances and decreased activity, known as cancer-related fatigue [1]. On the other hand, circadian disruption is a potential risk factor for the development and progression of some cancers, including malignant breast and gastrointestinal tumors [49].

In this study, we provide novel evidence that HCC in mice leads to significant changes at the level of the key component of the circadian system, the SCN. In particular, we see changes in neuropeptides and the activity of neurons and glial cells, as well as increased oxidative stress in SCN.

We previously showed a significant reduction in the overall locomotor activity, especially during the dark phase, reduced the amplitude of the activity rhythm, and reduced the neuronal activity in SCN during the light phase [37]. In accordance, mice with chemically induced HCC showed alterations in the locomotor activity rhythm [50]. These tumor-related changes in mice are similar to the symptoms of patients with ovarian, breast, liver, and colorectal tumors [51,52], indicating an effect of tumorigenesis on the circadian system, such as reduced activity, increased fatigability, and activity–sleep rhythm abnormalities. Furthermore, the SCN becomes less sensitive to the environmental photic cues in patients with liver cirrhosis [53], which is considered a premalignant condition [54]. Thus, HCC mice and patients with malignant tumors seem to share common changes in the circadian system. On the one hand, the SCN clockwork ensures the anticipation of the light and dark phases. On the other hand, the SCN clockwork is adaptively modulated by the light/dark information through a projection from the retina.

In SCN, neuropeptides, particularly VIP and AVP, are important for synchronization with the light/dark cycle to sculpt critical features of the sleep–wake cycle and for determining the period of circadian rhythms [10,17,18,55,56,57]. Both VIP- and AVP-immunoreactive SCN neurons play an important role in mediating the circadian output rhythm to subordinate oscillators in the brain, such as the paraventricular nucleus of the hypothalamus (PVN) which controls rhythms in the autonomic nerve system [58], secretion of hormones such as melatonin [58], and glucocorticoids [10] as well as the sleep–wake cycle [10]. These appear to be species-dependent differences in whether the AVP- and VIP-immunoreaction exhibit a time-of-day-dependent rhythm [59,60,61]. However, there is no question that the VIP+ neurons in the mouse SCN are activated by light and play an essential role in light entrainment of locomotor activity rhythm [17,18]. Importantly, both the VIP- and the AVP-IR are higher in the SCN of HCC mice than in the PB control mice. This is consistent with VIP and its VPAC receptors being upregulated in various tumors, including HCC [62], pancreas [63], lung [51], breast cancers [64], and glioma [65]. This upregulation is probably due to inflammatory processes associated with cancer development [62]. Although we cannot conclude from immunohistochemistry whether the higher staining intensity of neuropeptides reflects higher or lower secretory activity, a change in staining intensity definitely reflects a change in functional status.

We could show earlier that in the brains of HCC mice, the expression of genes encoding for proinflammatory cytokines is increased [36], supporting this hypothesis. Moreover, we could show earlier that the serum corticosterone levels are increased in HCC mice [37]. However, we cannot differentiate whether this is due to the inflammatory response or the disruption of the output of the SCN to the PVN or the hypothalamic–pituitary–adrenal (HPA) axis. Nevertheless, glucocorticoids not only provide time information for peripheral clocks but also modulate the VIP and AVP systems in the SCN [66]. Thus, increased immunoreaction of VIP and AVP in the SCN of HCC mice observed in this study might be due to the inflammatory response mediated by cytokines and/or glucocorticoids. In addition, metabolic changes associated with HCC can have a neuroactive effect (also see below).

Orexin is a neuropeptide exclusively produced by a specific subset of neurons in the lateral hypothalamus, and its dysfunction is associated with increased sleepiness and decreased activity [67,68]. Since there was no effect of HCC on orexin-IR in the lateral hypothalamus, a general impact on neuropeptide expression throughout the brain is unlikely.

VIP is important for robust rhythms in clock gene expression in the SCN and some peripheral organs [69] and for the entrainment of circadian rhythms to the light/dark cycle [10,18], presumably through CRE activation in the SCN [70], leading to c-Fos and clock gene expression [71]. However, chemogenetic activation of VIP+ SCN neurons attenuates the amplitude of circadian rhythms in the SCN, consistent with the concept that high levels of VIP lead to reduced synchronization between SCN cells [72]. Thus, the alteration of VIP and AVP systems in the SCN of HCC mice observed in this study might contribute to reduced precision in the alignment of activity to the light/dark cycle and the decreased amplitude in their activity rhythm previously reported in [37]. Consistently, the higher VIP-IR in HCC mice was associated with a decrease in c-Fos-IR (this study), similar to the decrease in pERK-IR [37] and an increase in Bmal1-IR (this study).

Bmal1 is essential for circadian rhythm generation [73] and the robustness of rhythmic activity in the light/dark cycle [12] In addition, Bmal1 is involved in the light-induced resetting of the SCN [13]. Bmal1-IR in the SCN shows no fluctuation depending on the time of day and is not affected by light [74]. Consistently, there was no effect of time on Bmal1-IR in the SCN of both groups. However, Bmal1 plays a critical role in circadian misalignment [75], and overexpression of Bmal1 leads to damping of SCN circadian rhythms [76]. Bmal1-IR was higher in the SCN of HCC mice compared to PB controls, suggesting a circadian misalignment and a mild overexpression. Thus, the lower levels of c-Fos and the higher levels of Bmal1 in the SCN of HCC mice might be due to the changes in the secretory activity of VIP+ neurons and might contribute to the reduced precision in the alignment of the activity rhythm to the light/dark cycle. Importantly, c-Fos is expressed in neurons only, while Bmal1 is also expressed in glia cells [14]. SCN astrocytes are important components of the rhythm-generating network, e.g., by regulating the balance between VIP and GABA [77]. They can initiate and maintain robust circadian activity rhythms and determine the circadian period [14,78]. Bmal1 might be involved in the regulation of the fine astrocytic processes that contribute to the chemical synapse [79]. The increased GFAP-IR in HCC mice indicates activation of astrocytes associated with the downregulation of their supportive function and upregulation of their proinflammatory function [80]. This might contribute to the alteration of neurotransmitter balance, reduced robustness of rhythmic locomotor activity, and also affect neurotransmission. Although there is no evidence for the involvement of microglia in the SCN circadian clock under physiological conditions [81], activation of proinflammatory microglia in SCN is associated with reduced rhythm amplitude in SCN slices [82]. The increased IBA-IR in HCC mice indicates activation of microglia accompanied by upregulation of their proinflammatory activity [83], which may contribute to impaired SCN function. Similarly, other peripheral malignant tumors, including breast cancer, trigger neuroinflammation and activation of glia cells [1,84] to secrete neurotoxic factors and chemokines and promote the recruitment of immune cells across the blood–brain barrier [80]. We hypothesize that cancer-related fatigue is related to impaired SCN function. Activation of astrocytes and microglia associated with liver diseases is also consistent with previous studies and may be a homeostatic response to protect brain tissue from higher levels of circulating cytokines and/or neuroactive/-toxic metabolites such as ammonia [85,86,87,88]. Both activated astrocytes and microglia can contribute to oxidative stress [89,90,91]. The increased IR for 8-OHdG-IR, a marker of DNA and RNA oxidative damage, in HCC mice, is consistent with higher oxidative/nitrosative stress and neuroinflammation in liver diseases [25,92,93]. Oxidation of DNA and RNA might contribute to transcriptional and posttranscriptional modulation of clock gene expression in the SCN of HCC mice [32]. This mechanism might be related to the effect of melatonin, which can act as a scavenger for radical oxygen species, on the consolidation of rhythmic locomotor activity in HCC mice [50]. In addition, our previous findings revealed that HCC triggered an increase and disruption in the rhythmic glucocorticoid in mice [37]. Similar results were observed in HCC [30] and metastatic colorectal patients [94]. This chronic increase in glucocorticoids stimulates the stress response pathway via the HPA axis, which in turn stimulates the immune system to secrete various cytokines and chemokines, leading to neuroinflammation [95].

However, in the hippocampus of HCC mice, the expression of inflammatory cytokines and clock genes was increased, and the number of proliferating cells in the neurogenic niche was decreased, while the IR for c-Fos, pERK, GFAP, IBA, and 8-OHdG was not affected [36,37]. This suggests a brain region-specific difference in the response of neurons and glia cells to HCC and a modulation of clock gene transcription and adult neurogenesis by tumor-associated factors independent of oxidative stress.

Importantly, dysregulation of the endogenous circadian clock due to consuming alcohol, high-fat diets, shift work, or jet lag misaligns the liver’s rhythmic metabolism and, thus, leads to liver pathologies such as HCC. A mice model with circadian malfunction due to chronic jet lag revealed more tendency to develop HCC in non-alcoholic fatty liver disease (NAFLD) due to disruption of the cell cycle components, including cell proliferation and apoptosis, and activation of oncogenic pathways such as WNT/β-catenin and TNFα-NF-κB in the liver [96].

This study has some limitations. The semi-quantitative measurements of the immunoreaction of relevant markers for neuronal and glial activity in the SCN have some limitations. While immunohistochemistry has the great advantage that it is restricted to the specific area under investigation and the signal is not “contaminated” by other brain areas as is the case in biochemical analyses of punch preparations, it cannot be determined whether the higher staining intensity of neuropeptides reflects higher secretory activity or storage of neuropeptides. This question can be solved only by additional invasive procedures such as microdialysis in future studies. It has been shown that HCC is more prevalent in males [97]; however, circadian rhythms are disrupted in both male and female HCC patients [30]. In addition, there is a sex-specific effect in a wide variety of circadian functions as well as in the response to chronodisruption [98]. Thus, it is still important to include HCC-bearing female subjects in future studies to identify sex-dependent differences in the response of the circadian system to HCC. It would also be of interest to detect the interaction between the development and progression of HCC and aging, which is correlated with dampened circadian rhythms, on the SCN function and the rhythmic behavior in further studies.

## 5. Conclusions

We propose a mechanism whereby cancer-related inflammation and/or neuroactive metabolites of liver dysfunction impair the circadian system at the level of the SCN. Our data suggest a double effect, on the one hand, with regard to neuronal activity and neuropeptides and, on the other hand, with regard to glial activity and oxidative stress. Both effects converge in a dysfunction of the molecular clockwork, which results in altered locomotor activity due to changed SCN rhythmic output. Our study contributes to the understanding of chronodisruption in cancer/liver diseases and could help develop corresponding therapeutic strategies to obtain better treatment outcomes and improve the quality of life of the patients.

## Figures and Tables

**Table 1:** Primary antibodies.

Antibody	Manufacturer	Concentration
Anti-VIP (rabbit, monoclonal, cat #4245)	BMA Biomedicals (Augst, Switzerland)	1:4000
Anti-AVP (rabbit, polyclonal, cat # AHP372)	BIO-RAD (Berkeley, CA, USA)	1:1000
Anti-Bmal1 (rabbit, monoclonal, cat #14268-1-AP)	Proteintech (Rosemont, IL, USA)	1:500
Anti-orexin (rabbit, monoclonal, cat # 16743)	Cells Signaling Technology (Danvers, MA, USA)	1:2000
Anti-IBA1 (rabbit, polyclonal, cat # 019-19741)	WAKO (Osaka, Japan)	1:2000
Anti-GFAP (mouse, monoclonal, cat # 556330)	BD Biosciences (Eysins, Switzerland)	1:500
Anti-8-OHDG (mouse, monoclonal, cat #AM03160PU-N)	ORIGENE (Herford, Germany)	1:1000
Anti-c-Fos (rabbit, monoclonal, cat #4384)	Cells Signaling Technology (Danvers, MA, USA)	1:1000

**Table 2:** Secondary antibodies.

Antibody	Manufacturer	Concentration
Anti-rabbit IgG Biotin (goat, cat # BA-1000)	Vector Laboratories (Burlingame, CA, USA)	1:500
Anti-rabbit IgG Alexa Fluor 488 (goat; cat # A-11036)	Molecular Probes (Eugene, OR, USA)	1:500
Anti-mouse IgG Alexa Fluor 568 (goat; cat # A-11031)	Molecular Probes (Eugene, OR, USA)	1:500

## Data Availability

The dataset supporting the conclusions of this article is available upon reasonable request to the corresponding author without restriction.

## Supplementary Materials

The following supporting information can be downloaded at: https://www.mdpi.com/article/10.3390/biomedicines12102202/s1, Figure S1. Liver inspection during autopsy; Figure S2. Anatomical localization of SCN and lateral hypothalamus (LH); Figure S3. Neuropeptides immunoreaction in SCN.

## Abbreviations

AVP: arginine vasopressin, CRF: cancer-related fatigue, DAB: 3,3′-diaminobenzidine, GFAP: fibrillary acidic protein, HCC: Hepatocellular carcinoma, IR: immunoreaction, 8-OHdG: 8-hydroxydeoxyguanosine, PB: Phenobarbital, PBS: phosphate buffered saline, PVN: paraventricular nucleus of the hypothalamus, RHT: retino-hypothalamic tract, SCN: suprachiasmatic nucleus, SEM: standard error of the mean, VIP: vasoactive intestinal peptide, ZT: Zeitgeber Time.
