# Supplementary material for: Hepatocellular Carcinoma in Mice Affects Neuronal Activity and Glia Cells in the Suprachiasmatic Nucleus
Source: Biomedicines. 2024 Sep 27;12(10):2202. doi: 10.3390/biomedicines12102202 (PMC11504045; doi:10.3390/biomedicines12102202)
Supplement: Supplementary file 1 [file biomedicines-12-02202-s001.zip › biomedicines-3207092-supplementary.pdf]

Supplementary figures:

Supplementary materials:

Figure S1

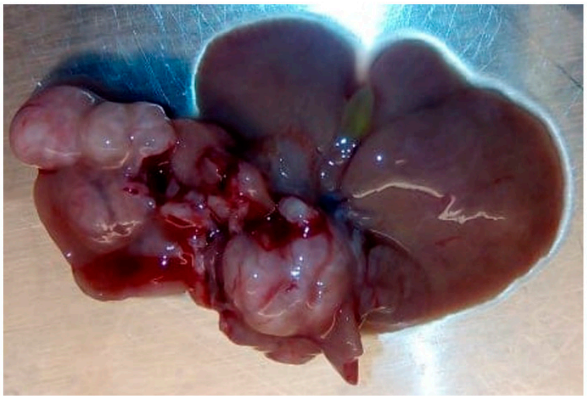

Figure S1. Liver inspection during autopsy. Representative image shows liver tumors in DEN+PB treated mice killed at the age of 8-10 months. The liver was macroscopically inspected and only DEN-treated mice with liver tumor were included in the HCC group. The livers of control mice did not show any macroscopic signs of pathology incidence and only the HCC-bearing mice of comparable lesion size were included for further analysis.

Figure S2

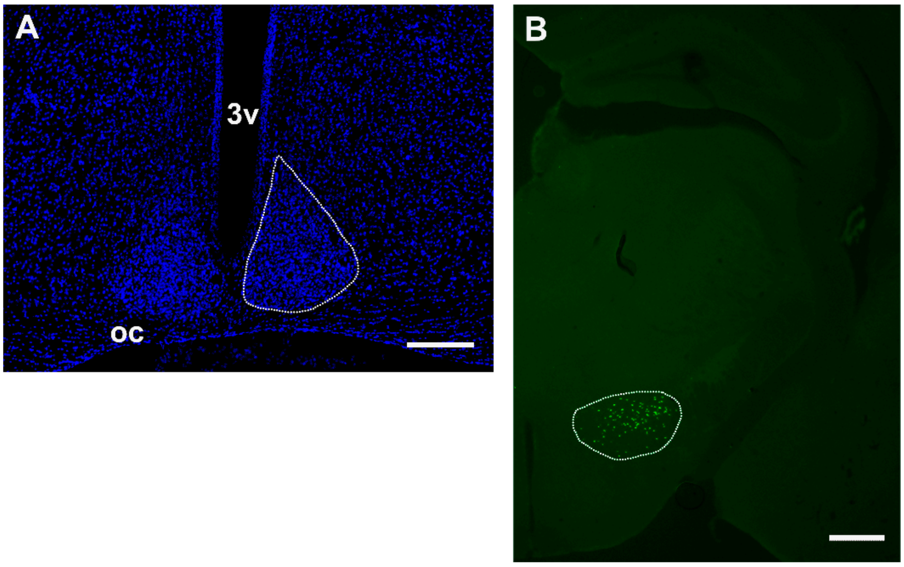

Figure S2. Anatomical localization of SCN and lateral hypothalamus (LH). Representative fluorescent photomicrographs show (A) delineated SCN characterized by DAPI-stained densely packed nuclei (blue) dorsal to the optic chiasma and on both sides of third ventricle. (B) Area of the lateral hypothalamus (delineated) showing orexin-immunoreactive neurons (green). 3V: third ventricle, OC: optic chiasma. Scale bar = 200 μm in (A). Scale bar = 1mm in (B).

Figure S3

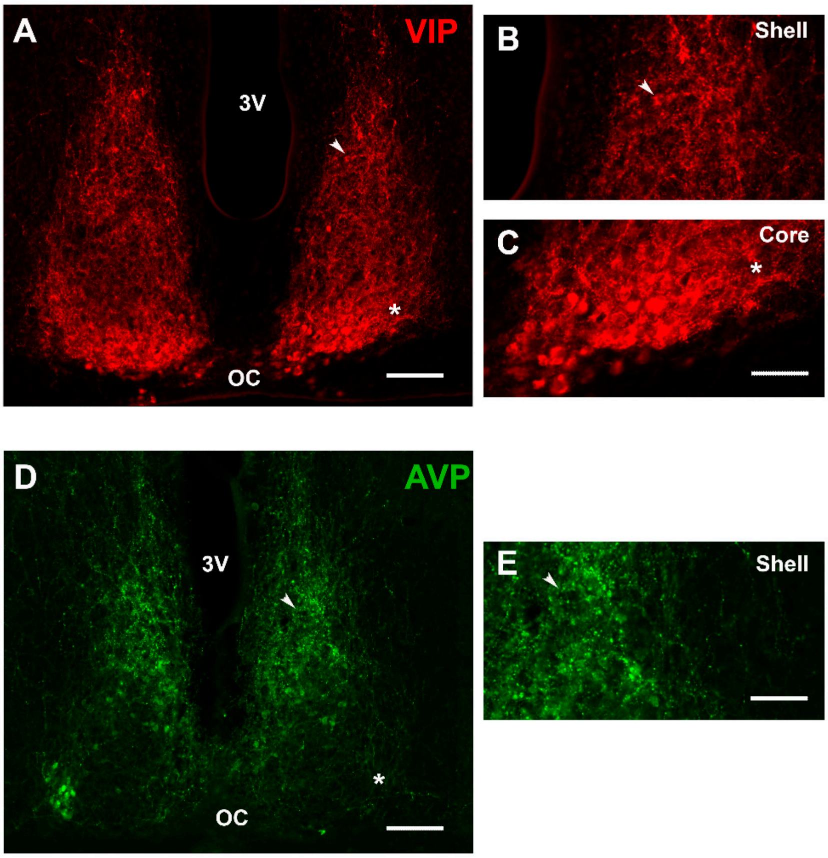

Figure S3. Neuropeptides immunoreaction in SCN. (A) Representative fluorescent photomicrograph shows VIP-immunoreaction (red) in SCN. High magnification photomicrograph shows VIP+ (B) axons (within shell region) and (C) cell bodies (within the core region). Representative fluorescent photomicrographs show (D) AVP-immunoreaction (green) in SCN. High magnification photomicrographs show (E) AVP+ axons and cell bodies (within shell region). Arrow-head indicates shell region. Asterisk indicates core region of the SCN. Scale bar = 100 µm in A, D. Scale bar = 50µm in B, C, E.
